# The effect of self-assessment on student competence in physiotherapy clinical training: a randomized controlled trial

**DOI:** 10.1186/s12909-023-04737-9

**Published:** 2023-10-19

**Authors:** Hammam Atrash, Michal Katz-Leurer, Gila Shahar

**Affiliations:** 1 Sackler Faculty of Medicine, School of Health Professions, Department of Physical Therapy, Tel- Aviv University, P.O. box 463, Dabburiya, Tel-Aviv Zip code 0016910 Israel; 2 Clalit Health Services, Horowitz Physical Therapy Clinic, Tel-Aviv, Israel

**Keywords:** Clinical training, Clinical Education, Physiotherapy, Self-assessment

## Abstract

**Background:**

Self-assessment is a method that allows students to reflect on and critically evaluate their performance, increases students’ involvement in learning, and improves academic achievement. In physiotherapy (PT) education, clinical training is a crucial component, guided by clinical educators (CEs), who assess and provide feedback, fostering student development. Limited research has investigated the impact of self-assessment on PT clinical training outcomes. This study aims to assess the effect of mid-term self-assessment during PT clinical training on students’ competence and on level of agreement between students’ self-assessment and CEs’ assessment at the end of the training.

**Methods:**

23 CEs and their 52 undergraduate PT students participated in the study. The students underwent eight weeks of clinical training in outpatient PT clinics in groups of two or three. For each group, one CE performed student assessment at the mid-term and the end of the training using the Assessment of Physiotherapy Practice (APP) form, an assessment tool used to evaluate clinical competence in PT clinical training. One student from each group was randomly assigned to join the intervention group (IG). These students completed a self-assessment process at the mid-term of the training. All students were asked to complete a self-assessment form at the end of the training.

**Results:**

The median CE’s evaluation score halfway through the training was 80 [50–96] and 91 [65–100] at the end of the training, with no significant differences between the two groups. The level of agreement between the student and CE’s evaluation at the end of the training was not significantly different between the groups (p = 0.05). It noted that students who scored themselves higher than their CEs tended to have lower APP scores than others, based on CEs’ assessment. These students were found to have less academic experience. Nevertheless, those from the IG improved significantly, based on the CE’s assessment, during the second half of the training, compared to the controls.

**Conclusion:**

The main finding of the present study is that student participation in self-assessment during PT clinical training is advantageous, mainly for individuals undergoing their initial clinical training and in the early stages of their academic studies.

## Background

Methods for training qualified medical professionals are continually developing over time [1]. Teaching paradigm has shifted from a passive learning approach to an active and cooperative one, emphasizing the student’s involvement in learning [2]. Active learning emphasizes the student’s commitment to the learning process and has been found to contribute to the progress of active lifelong learners with skills such as critical thinking, communication, and teamwork. These abilities play an essential role in therapy since professionals working in a diverse and dynamic healthcare system must keep current [3–6]. In addition, active learning can be an effective way to improve student satisfaction with the learning process [7].

A student’s evaluation is an invaluable part of the learning process. The most common source of information for evaluating a student’s learning process is performance assessment within three fields: knowledge, skills, and professionalism [1]. The evaluation process generally constitutes a complex challenge, specifically maintaining objectivity when relating to interpersonal skills such as behavior and communication [6]. Generally, the evaluation is the responsibility of the teacher. However, beyond responsibility for the learning process, active learning emphasizes student involvement through self-assessment [4].

According to Boud & Falchikov (1989), self-assessment relates to the student’s judgment in fulfilling the criteria or standards they set. A student who is not involved in establishing criteria but in making a judgment about his work is called self-marking. In this study, the self-assessment process does not include setting criteria and standards for work but does include a predetermined method for self-marking. However, the process does not only self-marking but also includes a discussion with the clinical educator (CE) about the student’s performance level and goal setting, so the term used is self-assessment.

The development of the ability for self-assessment has aroused great interest in the academic world since students’ involvement in the learning process is essential, as it improves academic achievement, contributes to professional development, improves overall performance, and assists the students to direct and focus their efforts to areas that require improvement [4]. A systematic review of self-assessment in medical education found that it can be a useful tool for identifying learner needs, improving learning, and impacting clinical practice. However, there is limited evidence and no clear consensus on the best methods for self-assessment. Self-assessment can empower learners by identifying strengths and weaknesses, setting goals for improvement, and tracking progress. It can also promote transformative learning engagement by encouraging learners to reflect on their own practice and seek out new opportunities to learn. For medical students and other healthcare professionals, self-assessment can help to ensure patient safety by providing them with a way to identify areas where they need to improve [8].

Despite the benefits stated above, there is insufficient knowledge about the reliability of the assessment within and between students. Furthermore, diverse student behavior patterns, especially in interpersonal skills such as communication, make standardizing more difficult [9, 10]. Additionally, accuracy is measured by comparing the student’s grade to the “gold standard”, which is usually the teacher’s evaluation. Here too, despite the evaluation criteria, a lack of uniformity among teachers still exists [10, 11].

Differences between student self-assessment and teacher’s or CE’s assessment may arise due to the extent of student engagement in the assessment process. This dynamic often results in elevated self-scores, especially among students facing challenges, whereas students excelling academically tend to provide more precise self-assessments. Enhanced accuracy in self-assessment fosters objectivity, encouraging constructive self-critique and driving progress, excellence, and professionalism [10].

Self-assessment is an evolving skill that changes according to content, relationships, and perspective. Professional training programs in the medical field emphasize the importance of self-assessment among students as a foundation for developing long-term learning skills and maintaining self-motivation in every profession [9]. In addition, self-assessment can help students to become more reflective learners, identify their strengths and weaknesses, and set goals for their improvement [12].

The educational process in the clinical environment differs from that in the classroom. The situation in the clinical field is more complex and creates a challenge for the student and the CE. Beyond theoretical knowledge and clinical expertise, the student must demonstrate skills like adhering to a schedule, communication skills with colleagues and patients, and empathy towards the patient. The CE must arrange that the student meets with a “real” patient while maintaining a suitable level of treatment quality and the patient’s welfare. At the end of the process, the CE evaluates the student and his performance. The autonomy and active learning process will likely improve the learning experience, clinical performance, and student achievements [13].

Clinical training is an essential component of health professional education. It provides students with the opportunity to apply their theoretical knowledge and professional expertise to real-world situations, and it can be a powerful catalyst for reflection. CEs can play a key role in facilitating reflection by evaluating students’ performance and providing feedback for their continuous development [3]. One of the evaluation form is the Assessment of Physiotherapy Practice (APP). This form is a comprehensive tool that covers seven essential aspects of the clinical training environment: professional behavior, communication, assessment, analysis and planning, intervention, evidence-based practice, and risk management [14].

A study examining the attitude of the CE regarding the basic requirements a student needs to begin clinical training found that self-assessment is a commonly required essential skill. This skill allows the student to take responsibility for professional and scientific development according to the student’s strengths and weaknesses [15].

Nevertheless, there is limited research on the effectiveness of self-assessment in clinical settings. This study aims to address this gap by examining the effects of self-assessment on student achievement and the level of agreement between students’ self-assessment and the CE’s assessment at the end of the training.

Therefore, the primary aim of this study was to examine the effect of self-assessment at the mid-term of PT clinical training on the student’s achievement and the level of agreement between students’ self-assessment and the CE’s assessment at the end of the training.

The secondary aim was to describe the characteristics of students who tended to overestimate their achievements.

## Methods

### Study design

The study is a randomized controlled trial.

### Setting

A PT bachelor’s degree program in Israel lasts four years and consists of clinical training in various fields with around 1,000 credit hours. Clinical training takes place in public hospitals and outpatient clinics. Students usually start clinical training in their third and fourth years of studies. Students in the clinical training are instructed by physiotherapists who additionally were trained to be CEs. Students are closely supervised by their CE most of the time, who oversees their work, provides them with feedback, and monitors their progress. Most clinical training lasts eight weeks (35 credit hours per week), and the CE assesses the student’s performance while holding two major assessment meetings – mid-term and final. The mid-term assessment meeting takes place during the fourth week of the training. Using the APP form, the CE explains the reason for each score to the student. In addition, the CE gives the student overall feedback while setting improvement goals. The final assessment takes place during the last week of training and includes a concluding assessment score by the CE, along with general feedback. In this study, students in the intervention group (IG) were asked to assess their performance before the mid-term assessment meeting using the APP and discuss it with their CE, providing them with an opportunity for improvement during the rest of the clinical training. Students in the control group will not undergo self-assessment.

### Sample size

The sample size calculation was based on Metz et al. (2017) study results and on the assumption that a moderately strong association (r = 0.6) will be seen at the end of the clinical training between students’ self-assessment score and CE score in the IG, while no such association will be seen in the control group (CG). Thus, with a type I error of 5% and a power of 80%, the required sample size was 40 students.

### Participants

A total of 23 CEs and their 52 students joined the study. Each student was enrolled for eight weeks of musculoskeletal clinical training in one of the Clalit Health Services outpatient clinics in three main regions in Israel (Central, Jerusalem, and Tel Aviv). Clinical education within Clalit Health Services have undergone a standardized training procedure across different departments. Students were included in this study if they studied for a bachelor’s degree in PT at Israeli higher education institutions during the 2018–2019 academic year. Only certified physiotherapists with a group of at least two students in training were included in the study. Each CE participated in the study only once. The Ethics Committees of Clalit Health Services and Tel Aviv University approved the study. Written consent from the students and the CEs was obtained prior to participation in the study.

### Randomization

After completing the mid-term assessment form, the CE randomly assigns students to the IG or CG using sealed envelopes.

### Assessment tool

Students’ achievements were evaluated using the APP form, a standardized instrument to evaluate clinical competence in PT clinical training. The APP lists 20 items that evaluate students’ performance in seven domains: professional behavior, communication, diagnostic assessment, analysis and planning, intervention, evidence-based practice, and risk management. A rating scale from 0–4 is used, where a higher value indicates a better performance level. The rating scale also includes a “Not Assessed” option, used if the student did not have the opportunity to demonstrate the item. The total score of the APP is the sum of the 20 items’ scores and ranges from zero to 80. The APP also includes a global rating scale of “Not adequate,” Adequate,” Good,” and “Excellent,” indicating the student’s overall performance. The global rating scale is not included in the total score [14].

The APP is reliable and valid with a percentage of agreement that ranges from 56% (Item number 19, Evidence-based practice) to 83% (Item number 20, Risk Management), and across all domains with an average of 70%. The intraclass correlation value of the total score is 0.92, with a confidence interval of 95% ranging from 0.84 to 0.96. The analysis of the APP content validity using factor analysis and Rasch analysis indicates that two dimensions are being measured by the APP: professional behavior and clinical performance [14, 16, 17].

The APP has been translated into Hebrew and found to be reliable, with a percentage of agreement ranging from 36.6% (Item number 19, Evidence-based practice) to 83.3% (Item number 1, Professional behavior) with an average of 63%. The intraclass correlation value of the total average score is 0.88, with a confidence interval of 95% ranging from 0.77 to 0.94 [18].

### Study procedure

The study was held in Clalit Health Services outpatient clinics. At the beginning of the training, the first author (HA) visited each clinic. He gave the CE and the students an explanation of the study’s goals and methods. Participants’ characteristics and written consent were obtained from all the CE and students. The CE received a research envelope that included the APP forms and a randomization sealed envelope.

During week four of the training, the CE conducts a mid-term assessment meeting. The CE was asked first to evaluate all students using the APP and only then to proceed with randomization. The CE let each student choose one of the sealed envelopes. Only one student received an envelope with an intervention sign. This student performed a self-assessment using the APP form before the mid-term assessment meeting. During the meeting, students receive feedback and assessment regarding their performance based on CE evaluation using the APP form. Students in the IG received mid-term assessments taking into consideration the assessment form filled out by the CE and the self-assessment form filled out by the student himself. Students in the CG received mid-term assessments based on the APP form filled out by the CE. These APP files were placed in an envelope and sent to the first author.

At the end of the clinical training, during week 8, CE asked to evaluate all students. In addition, all the students in IG and CG were asked to perform self-assessments using the APP form. These assessment forms were not included in the final assessment meeting as they were explicitly done for the study and not for the official assessment that was sent to the educational institution. These APP files were again placed into an envelope and sent to the first author.

The CE and the students were asked not to calculate the total score of the APP.

Flowchart 1 presents the study procedure in each clinical training.

**Flowchart 1:**
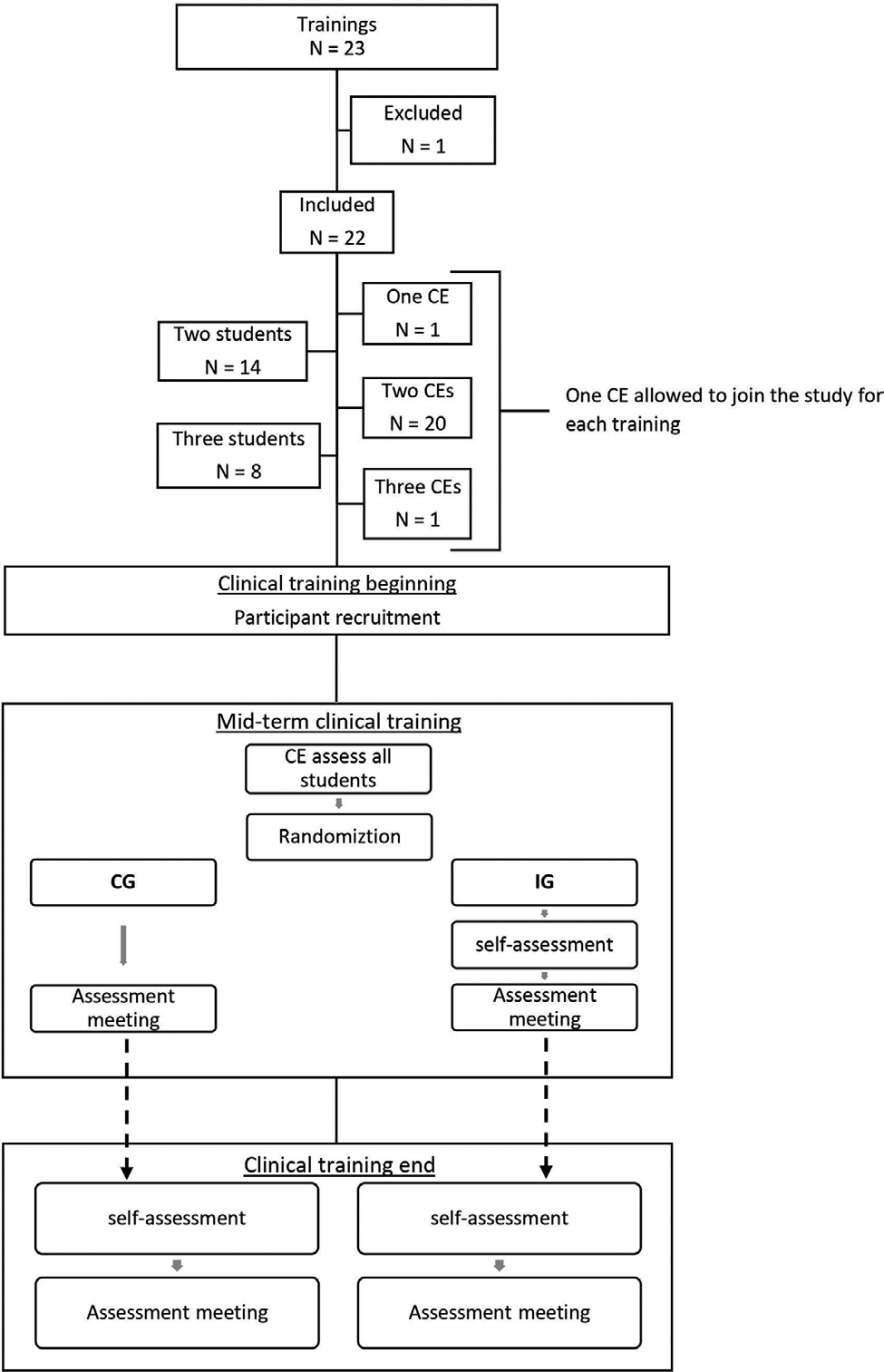
Clinical trainings recruitment and study procedure in each clinical training

### Statistical analysis

Participants’ characteristics, by study groups, are presented by frequencies and percentages for nominal variables and median and minimum-maximum for ordinal or ratio variables. Differences between groups were tested by 𝛘2, and Mann Whitney U test, respectively. Spearman ranks correlations and scatterplots show the association between students and CE scores. Each group was divided into two sub-groups based on their first self-assessment results (IG- mid-term, CG- end of the clinical training): Students who rate themselves either more than the CE (overestimate) or less (underestimate) than the CE. Differences between groups and sub-groups were based on the Mann-Whitney U test, and the Wilcoxon test showed change over time within the group. A test was defined as significant for p-value < 0.05, and data analysis was performed using SPSS-25 software.

## Results

### Participants’ characteristics

A total of 23 CEs and their 52 students participated in this study. One CE and his two students were excluded from the study after he gave both a self-assessment to perform during the mid-term period of clinical training. The CEs, half of whom are men, have a median age of 40 years (with a range of 29–63 years), a median experience as physical therapists of 13 years (with a range of 3.5–33), and a median of years of experience in clinical education of 5 years (with a range of 1–28). 13 have a bachelor’s degree in PT, and nine have a master’s degree. Students’ characteristics are presented in Table 1. Of the 50 students who were included, 22 were assigned to the IG. 72% of the students were female, 40% were in their first clinical training session, and 46% were in their fourth and final academic year. No significant differences were noted in students’ demographic and academic characteristics (p > 0.05).

**Table 1:** Student characteristics

Characteristic		Total (N = 50)	IG (N = 22)	CG (N = 28)	p-value
Gender (n)	Male	14	7	7	0.59
	Female	36	15	21	
Age (years)		26 [21–36]	26[23–31]	26[21–34]	0.87
Clinical training session (n)	1st	20	9	11	0.98
	2nd − 4th	30	13	17	
Academic year (n)	3rd	27	13	14	0.52
	4th	23	9	14	

Values in the table are number, median [Min-Max], p-value is based on 𝛘2, Mann Whitney U test

### Student achievements

Mid-term and final CEs’ APP total score and the change over time (Delta Score, ∆S) are presented in Table 2. There were no significant differences in total scores between groups at the mid-term and the end of the clinical training. Nevertheless, significant improvements in APP total scores over time were noted in both groups.

**Table 2:** Mid-term and final total APP score of ’CEs’ assessments and ∆S

CE assessment	Total (N = 50)	IG (N = 22)	CG (N = 28)	p-value* (between groups)
Mid-term score (/80)	64 [40–77]	63 [40–73]	64 [51–77]	0.28
End score (/80)	73 [52–80]	73 [59–78]	73 [52–80]	0.51
∆S	8 [(-8)-20]	10 [1–20]	7 [(-8)-10]	0.21
P-value# (within the group)		< 0.01	< 0.01	

Values in the table are median [Min-Max], p-value* is based on Mann Whitney U test, p-value# is based on the Wilcoxon test

### Level of agreement

**Fig. 1:**
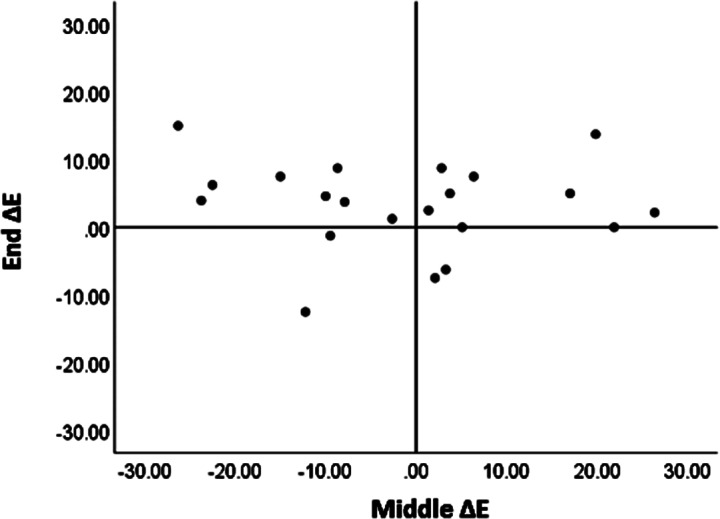
The ∆E per student in IG at mid-term and at the end of the training

A non-significant association was found at the mid-term of the clinical training in the IG between CEs’ assessment total score and students’ self-assessment total score (Spearman correlation coefficient (r_s_=0.15, p-value = 0.5) (Data not present in table). Figure 1 illustrates the distribution of differences in scores between the CE’s assessment and the student’s self-assessment (Delta Evaluation, ∆E) at the mid-term and end of the IG clinical training. The median of the ∆E at the mid-term of the training was 0.6, ranging from (-26) to 26. Eight of the students overestimated themselves compared to the CE scores at the mid-term and underestimated themselves compared to the CE scores at the end of the clinical training.

A significant positive moderately strong association was found between CE’s assessment and student’s self-assessment scores at the end of the clinical training; r_s_=0.53 (p-value = 0.01) in the IG and r_s_=0.49 (p-value = 0.02) in the CG. The strength of the association was not significantly different between groups. The median of the ∆E in the IG was 3.2 points [(-10)-12] (p = 0.03), while in the CG, it was 0 [(-14)-15] (p = 0.65), with no significant difference between groups (P = 0.10) (Data not present in table).

The initial self-assessment results were used to divide each group into two sub-groups. In the IG, 11 students who overestimated their abilities at the mid-term were placed in one subgroup, while the remaining 11 were placed in another subgroup. Similarly, in the CG, 11 students who overestimated their abilities at the end of the clinical training were placed in one subgroup, and the remaining 17 were in the other. This resulted in a total of four sub-groups.

The combined effect of group classification (IG vs. CG) and self-assessment agreement (overestimation vs. underestimation) is presented in Table 3. A significant improvement in APP total scores over time was noted in each subgroup. Moreover, a significant difference was noted in the CEs’ scores between the underestimated and overestimated students at the mid-term of clinical training, with the overestimated students tending to have lower CE scores. At the end of the training, no difference in CE scores was noted in the IG group, whereas the subgroup of overestimating students in the CG still shows lower CE scores. Overestimating in the IG showed the highest ∆S, with a median of 13 ranging from two to 20.

**Table 3:** Mid-term and final CEs’ APP scores by groups (IG vs. CG) and subgroups

CE assessment	Total (N = 50)			IG (N = 22)			CG (N = 28)		
	CE ≥ Student (N = 28)	Student > CE (N = 22)	p-value*	CE ≥ Student (N = 11)	Student > CE (N = 11)	p-value*	CE ≥ Student (N = 17)	Student > CE (N = 11)	p-value*
Mid-term (/80)	65 [51–76]	62 [40–67]	< 0.01	65 [53–73]	61 [40–66]	0.19	66 [51–76]	63 [56–67]	0.02
End (/80)	73 [59–80]	72 [52–78]	0.30	71 [59–77]	73 [60–78]	0.69	73 [67–80]	71 [52–77]	0.08
∆S	7 [0–16]	11 [(-8)-20]	< 0.01	7 [0–13]	13 [2–20]	0.01	7 [1–16]	10 [(-8)-15]	0.22
P-value#	< 0.01	< 0.01		< 0.01	< 0.01		< 0.01	0.01	

Values in the table are median [Min-Max], p-value* is based on Mann Whitney U test, p-value# is based on the Wilcoxon test

Students’ characteristics according to under and overestimating are presented in Table 4. Students in their first year of clinical training showed a significantly higher proportion of overestimating (59% vs. 25%, p-value = 0.01) than those in their third year (72% vs. 39%, p-value = 0.02); no significant relationship was found in the CE’s characteristics.

**Table 4:** Student characteristics according to estimation level at first self-assessment

Characteristic		Student > CE (N = 22)	CE ≥ Student (N = 28)	p-value
Gender (n)	Male	11	13	0.80
	Female	11	15	
Age (years)		26 [22−31]	26 [21−34]	0.81
Training session (n)	1st	13	7	0.01
	2nd − 4th	9	21	
Academic year (n)	3rd	16	11	0.02
	4th	6	17	

Values in the table are number, median [Min-Max], p-value is based on 𝛘2, Mann Whitney U test

## Discussion

This study examines the effect of self-assessment on the student’s achievement during the mid-term of PT clinical training and the level of agreement between students’ self-assessment and CE’s assessment at the end of the training. No significant difference was noted in CE’s final APP score between groups; both groups showed a significant improvement in their outcomes (∆S). The level of agreement between CE’s assessment and the student’s self-assessment at the end of the training was not significantly different between groups. In addition, students who tended to rate themselves with a higher score than the CE achieved significantly lower scores in the CE’s assessment than other students. These students were generally in an earlier stage of academic and clinical education. Of them, students in the IG changed their perception, assessed themselves the same as the CE or even less, and achieved better scores at the end of the training. No effect of CE’s characteristics on students’ scores and the level of agreement was found.

The primary hypothesis of this study was that a mid-term self-assessment process through PT clinical training would significantly improve students’ final scores and accuracy compared to the CG. This hypothesis was based on previous studies where student achievement changes were seen following self-assessment procedures [13, 19–22]. In the current study, no such effect was found.

What might explain the difference between this study and previous studies’ outcomes? First, in previous studies, the focus was mainly on students’ technical performance [13, 19–22]. According to Abiad (2018), self-assessment may increase students’ confidence when performing assignments. It may be that the self-assessment process has a different impact on professional behavior and clinical performance than technical performance.

A second possible explanation may be associated with the amount of experience needed for performing an effective self-assessment. In Abiad (2018) study, students were trained to perform self-assessments for an entire year, significantly improving their self-assessment accuracy. A review by Boud & Falchikov (1989) concluded that graduate students, and students who have been in academic studies for a relatively long period, more than three years, had a more accurate self-assessment when compared to junior students. In this study, senior students who had previous experience with the APP, fourth-year students, or students that underwent at least one clinical training were more accurate in their self-assessment than junior students, third-year students or those with only one clinical training. Information was gathered concerning students’ academic experience in PT, not about other academic experiences in other fields.

A possible methodological explanation for the fact that no difference was found between groups in the final scores is a “ceiling effect”. Both groups present high median scores in the mid-term assessments; according to these grades, the improvement range- up to 80- is limited. In addition, both groups received high scores at the end of the training (median score 73/80).

Students in the IG who overestimated their abilities at the mid-term demonstrated a significant improvement as assessed by the CE at the end of the training. In addition, they rated themselves lower than the CE at the end of the training. This change can be attributed to integrating the self-assessment process in the mid-term assessment meeting.

As indicated in a previous review [8], self-assessment has the potential to aid learners in the identification of strengths and weaknesses, facilitating the establishment of improvement goals, and enabling the tracking of progress. In this study, students in the IG were asked to evaluate their own performance using the APP before their mid-term assessment meeting. This may have helped them identify their strengths and weaknesses and to set goals for improvement. They then met with their CE to discuss their self-assessment and to get feedback on their performance. The CE reviewed the student’s performance over the initial half of the training, factoring in both the app-generated evaluation and the student’s self-assessment. This allowed the CE to have a more holistic understanding of the student’s performance and to provide more targeted feedback. In contrast, students in the control group only met with their CE to receive feedback based only on the CE’s observations which may have resulted in a less comprehensive and less accurate assessment of the student’s performance.

The main strength of this study is that randomization took place only after the completion of student evaluations by the CE in the mid-term. Another strength of this study is the fact that it was conducted under clinical settings.

### Limitations

A possible limitation of this study is associated with its external validity. A review by Mccallum, Reed, Bachman, & Murray (2016) examined the effect of CE’s characteristics on CE’s and student’s performance, either by the CE’s self-perception or by the student’s perception. The result was not unequivocal, but some studies have indicated a positive effect of work seniority, CE’s seniority, academic degree, CE course, and more. In this study, no effect of the CE’s characteristics was found on student scores and the level of agreement. A possible reason for these findings is that the clinical training process in Clalit Health Services has been standardized, which leads to uniformity between the various CEs’, programs, and structure of the clinical training. Another possible reason is that all the CEs’ who participated in this study underwent a clinical training course. Previous studies have examined the effect of a training course and found a secondary effect of the course on the CE’s performance and student achievement [23–25]. In addition, it would have been worthwhile to assess which of the sub-topics of the APP contributes more to the differences in agreement on the achievement and the overall score, but this study lacks the statistical power required for this question.

## Conclusion

Based on the results of this study, the self-assessment process during the mid-term of PT clinical training is essential among beginning students in their clinical training process and academic careers. In addition, it was found that familiarity with the self-assessment process can positively affect the learning process. Therefore, self-assessment instruction, in general, and the use of an assessment form in particular, may lead to accuracy in self-assessment and possible improvement in achievement. In the next step, the integration of a structured self-assessment process during a mid-term training assessment should be examined. In addition, assessing the effect of the self-assessment process in other clinical education fields in PT would be worthwhile.

## Data Availability

The datasets used and/or analyses during the current study available from the corresponding author on reasonable request.

## List of abbreviations

APP: Assessment of Physiotherapy Practice
CE: Clinical Educator
PT: Physiotherapy
IG: Intervention Group
CG: Control Group
∆S: Delta Score
∆E: Delta Evaluation

## References

[1] Notzer N, Abramovitz R (2011). How to prepare a medical student to become a physician?. Harefuah.

[2] Johnson DW, Johnson RT, Smith KA (1998). Active learning: Cooperation in the College Classroom.

[3] Mann K, Gordon J, MacLeod A (2009). Reflection and reflective practice in health professions education: a systematic review. Adv Health Sci Educ.

[4] Mentz E, de Beer J, Bailey R, Bergamin PB, Bosch C, Toit A, du, Goede R, Golightly A, Johnson DW, Johnson RT, Kruger C, Laubscher D, Lubbe A, Olivier J, van Zyl S. Self-Directed Learning for the 21st Century: implications for higher education. AOSIS; 2019.

[5] Prince M (2004). Does active learning work? A review of the Research. J Eng Educ.

[6] Zimmerman S, Byram Hanson D, Stube J, Jedlicka J, Fox L. Using the power of Student Reflection to Enhance Professional Development. Internet J Allied Health Sci Pract. 2007.

[7] Hyun J, Ediger R, Lee D (2017). Students’ satisfaction on their learning process in active learning and traditional classrooms. Int J Teach Learn High Educ.

[8] Colthart I, Bagnall G, Evans A, Allbutt H, Haig A, Illing J (2008). The effectiveness of self-assessment on the identification of learner needs, learner activity, and impact on clinical practice: BEME Guide no. 10. Med Teach.

[9] Dauenheimer DG, Stahlberg D, Spreeman S, Sedikides C (2002). Self-enhancement, self-verification or self-assessment? The intricate role of trait modifiability in the self-evaluation process. Revue Int de Psychologie Sociale.

[10] Sedikides C (1993). Assessment, enhancement, and verification determinants of the self-evaluation process. J Personal Soc Psychol.

[11] Boud D, Falchikov N (1989). Quantitative studies of student self-assessment in higher education: a critical analysis of findings. High Educ.

[12] Evans AW, McKenna C, Oliver M (2002). Self-assessment in medical practice. J R Soc Med.

[13] Bolívar-Cruz A, Verano-Tacoronte D, González-Betancor SM. Is University Students’ Self-Assessment Accurate? Sustainable Learn High Educ. 2014:21–35.

[14] Dalton M, Davidson M, Keating JL (2012). The Assessment of Physiotherapy Practice (APP) is a reliable measure of professional competence of physiotherapy students: a reliability study. J Physiotherapy.

[15] Eva KW, Regehr G. Self-Assessment in the Health Professions: a reformulation and research agenda. Acad Med. 2005;80. 10.1097/00001888-200510001-00015 16199457

[16] Dalton M, Davidson M, Keating J (2011). The Assessment of Physiotherapy Practice (APP) is a valid measure of professional competence of physiotherapy students: a cross-sectional study with Rasch analysis. J Physiotherapy.

[17] Reubenson A, Ng L, Gucciardi DF (2020). The Assessment of Physiotherapy Practice tool provides informative assessments of clinical and professional dimensions of student performance in undergraduate placements: a longitudinal validity and reliability study. J Physiotherapy.

[18] Schwartz D, Jacob T (2019). Establishing the reliability of a Tool for assessing israeli physical therapy students. J Phys Therapy Educ.

[19] Andrade L. Students as the definitive source of formative assessment: academic self-assessment and the self-regulation of learning. Handbook of Formative Assessment. Routledge; 2010:102–17.

[20] Abiad RS. Effect of self-assessment training in preclinical endodontic courses on the clinical performance of undergraduate dental students. Australasian Med J. 2018;11(5).

[21] Panadero E, Brown GTL, Strijbos J-W (2016). The future of Student Self-Assessment: a review of known unknowns and potential directions. Educational Psychol Rev.

[22] Metz MJ, Durski MT, ’Malley O’Malley DeGaris M, Daugherty TC, Vaught RL, Cornelius CJ, Mayfield TG (2017). Student Self-Assessment of Operative Dentistry Experiences: a Time-Dependent Exercise in Self-Directed Learning. J Dent Educ.

[23] McCallum CA, Reed R, Bachman S, Murray L (2016). A systematic review of physical therapist clinical instructor demographics and key characteristics: impact on Student Clinical Education Experiences. J Phys Therapy Educ.

[24] Housel N, Gandy J (2008). Clinical instructor credentialing and its Effect on Student Clinical Performance Outcomes. J Phys Therapy Educ.

[25] Housel N, Gandy J, Edmondson D (2010). Clinical instructor Credentialing and Student Assessment of clinical instructor effectiveness. J Phys Therapy Educ.

